# Supplementary material for: Deep reinforcement learning can promote sustainable human behaviour in a common-pool resource problem
Source: Nat Commun. 2025 Mar 22;16:2824. doi: 10.1038/s41467-025-58043-7 (PMC11929920; doi:10.1038/s41467-025-58043-7)
Supplement: Supplementary file 1 — Supplementary Information [file 41467_2025_58043_MOESM1_ESM.pdf]

Supplementary Materials for:

Deep reinforcement learning can promote sustainable human behaviour in a common-pool resource problem

Raphael Koster[1]*, Miruna Pîslar[1]*, Andrea Tacchetti[1], Jan Balaguer[1], Leqi Liu[1,2], Romuald Elie[1], Oliver P. Hauser[3], Karl Tuyls[1], Matt Botvinick[1,4], Christopher Summerfield[5].

[1] Google DeepMind, London, UK.
[2] Princeton University, Princeton, USA.
[3] University of Exeter, Exeter, UK.
[4] Yale Law School, Yale University, New Haven, USA.
[5] University of Oxford, Oxford, UK.

* author equal contribution
Correspondence: rkoster@google.com, mirunapislar@google.com

Supplemental Results

In main text Figure 2A, we observed that games played under the equal baseline led to lower surplus than the other conditions (equal < mixed, $z = 4.58$, $p < 0.001$; equal < proportional, $z = 5.28$, $p < 0.001$; all Wilcoxon rank sum tests unless otherwise specified), whereas games played under the proportional baseline led to higher Gini coefficient (over total player surplus) than other conditions (proportional > mixed, $z = 5.89$, $p < 0.001$; proportional > equal, $z = 4.76$, $p < 0.001$). In Fig. 2A (right panel) we show aggregate surplus and Gini coefficient for each mechanism in the 40 games played with human participants in Exp.1. The equal (blue dots) and mixed (purple dots) conditions yield low surplus and low Gini (~0.1; because surplus is uniformly low), whereas proportional (red dots) has a higher surplus but incurs a much higher Gini of just under ~0.4. When we computed the fraction of games that were sustained to the final (40th) round, we found that 60% of proportional games were sustained with at least one player, but none with all four players; by contrast, in mixed or equal conditions, where games were either sustained by everyone or not at all, 30% and 5% of games finished with all four players still active. Thus, our baseline mechanisms were not successful at encouraging sustained reciprocation from human players.

Fig. S1

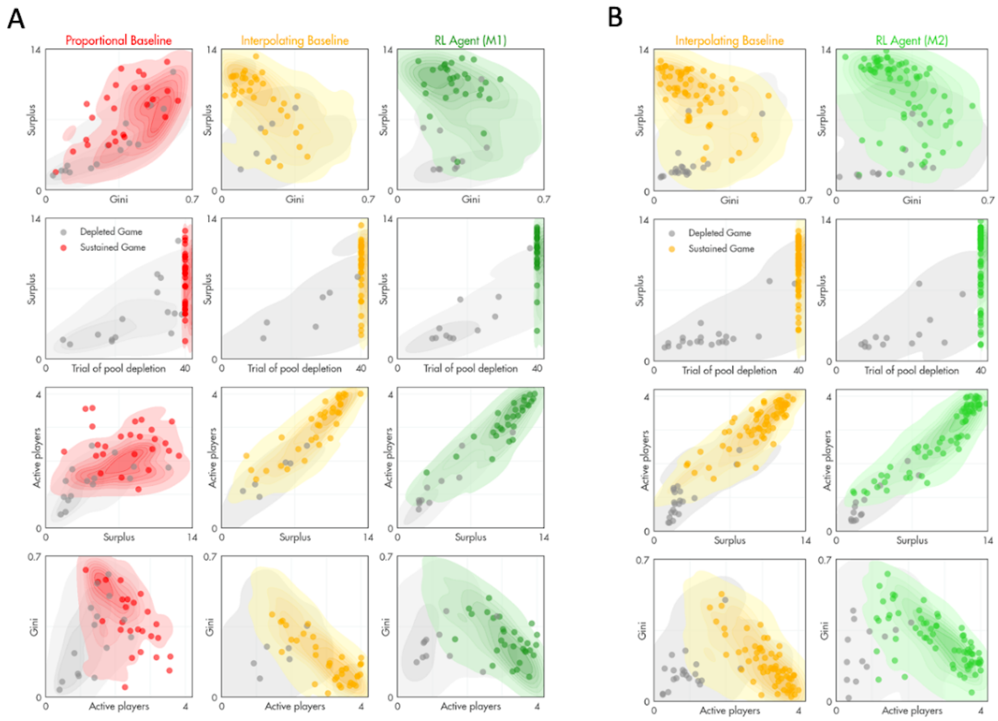

Figure S1. Game metrics from Exp.2 and 3. A. Correspondences between predicted outcomes (from virtual players, shading) and observed outcomes in Exp.2 (dots) for the proportional (red) and interpolating (yellow) baselines and the RL agent (green). Shown separately for games that were sustained to the end by at least one player (colours) and those where the pool was exhausted prematurely (grey). **B.** The same for Exp. 3. Equivalent to Fig. 2B.

Fig. S2

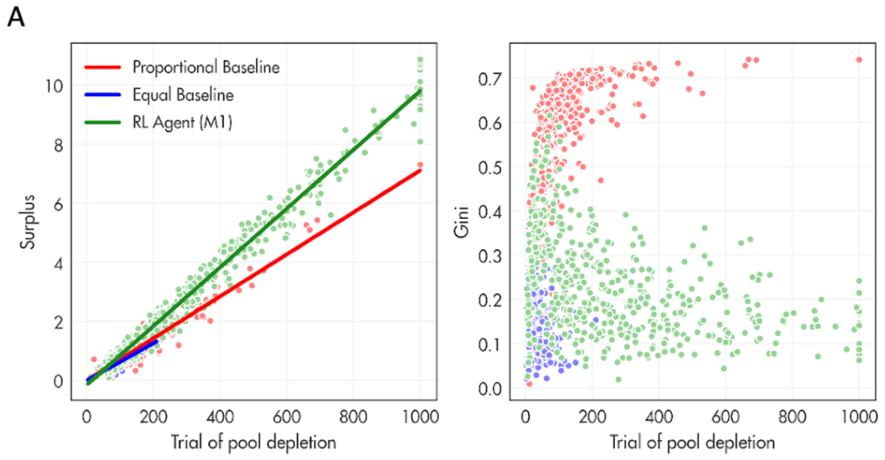

Figure S2. Simulation of longer episodes. A. Plots of surplus (left panel) and the Gini coefficient (right panel) against the trial on which the pool was depleted (x-axis) for 512 games unrolled for 1000 rounds each using virtual players. The red, blue and green dots are individual games played with the proportional baseline, equal baseline, and RL Agent (M1) mechanisms. The RL Agent can sustain the pool for an average of 271 ± 251 rounds, compared to 32 ± 28 and 105 ± 102 for the Equal and Proportional baselines, respectively.

Fig. S3

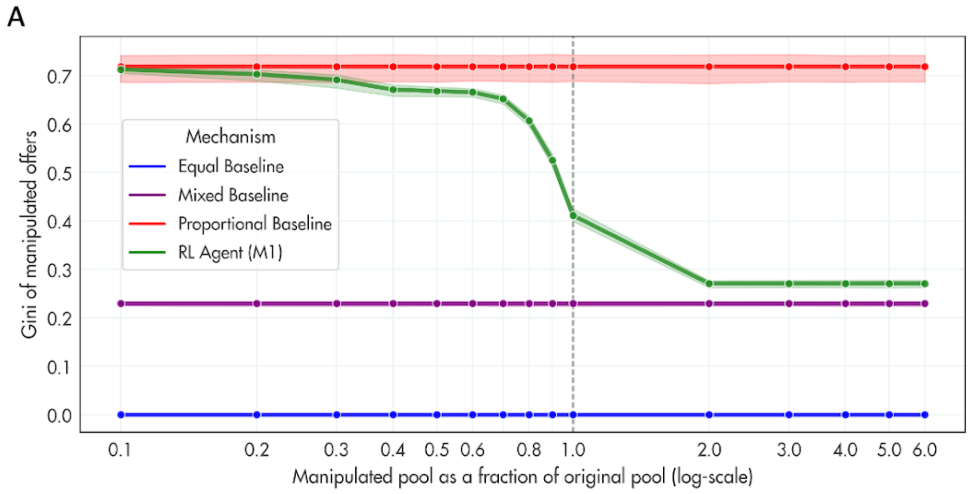

Figure S3. Analysis of agent policy. A. Results of an intervention experiment in which we systematically provided misleading information to the RL agent about the pool size. We systematically multiplied the estimate of the pool size by a coefficient (x-axis) between 0.1 and 6, and plotted the Gini coefficient of the average offer the RL agent (green line) made to players (y-axis). For reference we show the average Gini of the offer made under three baseline conditions from Exp.1 (blue, purple and red lines). When the pool was lowered by 90% (leftmost point), the RL agent behaved similarly to the proportional baseline. Whe pool size was increased, it behaved in a more egalitarian fashion.

Fig. S4

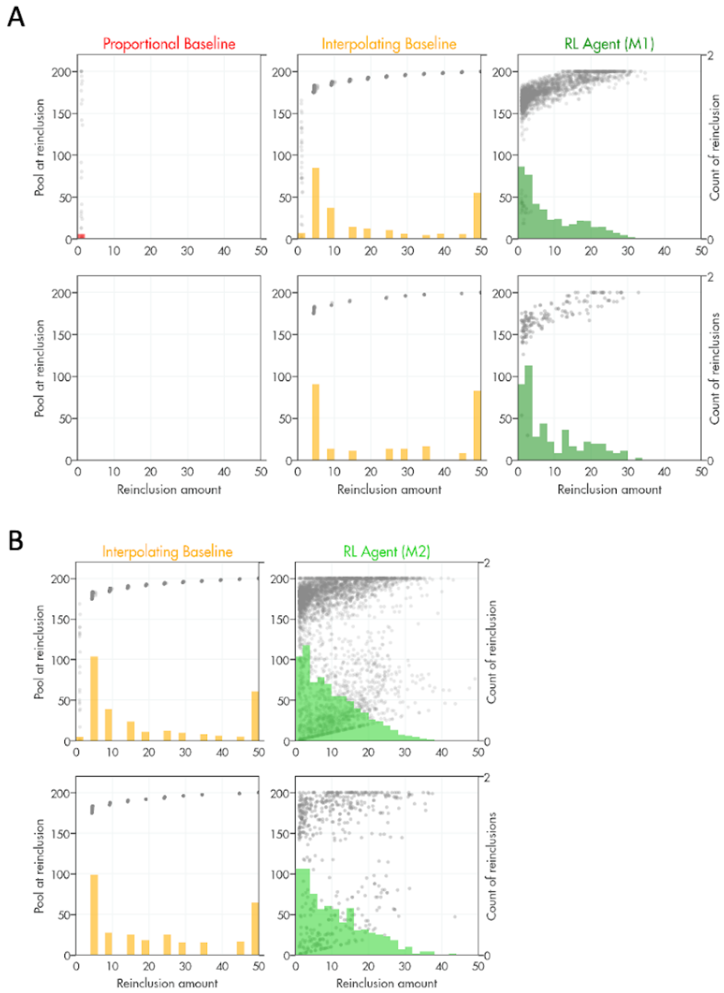

Figure S4. Reinclusion in Exp. 2 and 3. A. On each plot, the coloured bars show the distribution of offers made on the "reinclusion" trial (following one or more offers of < 1) by the proportional, interpolating and RL agent in Exp.2. There are no bars for the proportional mechanism because it cannot reinclude. Grey dots show individual reinclusion events, plotting the amount against the pool size at this event (y-axis left). In all plots the top row shows data from virtual players and the bottom row shows human data. **B.** The same data for the interpolating baseline and RL agent in Exp.3.

Fig. S5

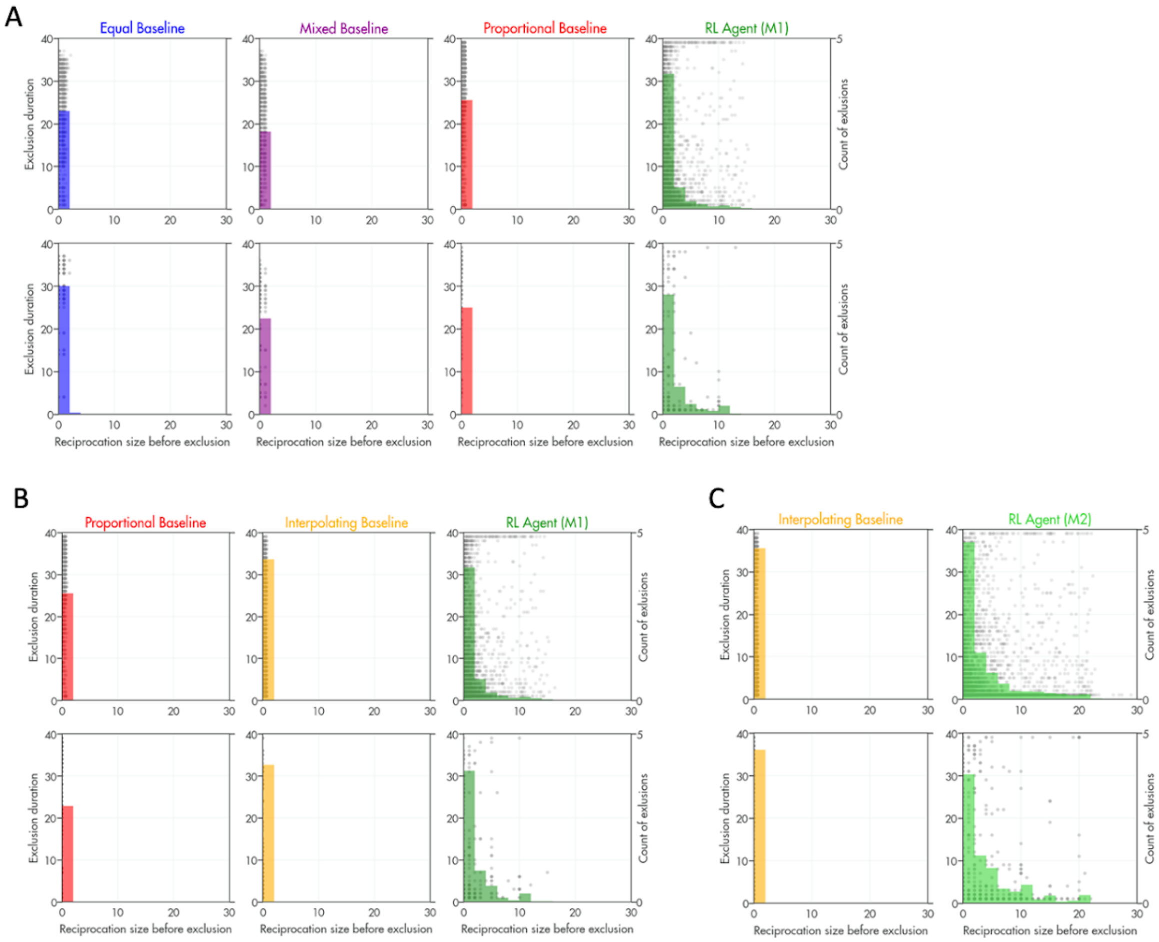

Figure S5. Exclusion durations. A. Duration of exclusion by reciprocation size at the time of exclusion for each mechanism in Exp. 1. The left y axis shows the duration of the exclusion in trials. The x axis shows the reciprocation size of the player that led to their exclusion. The histogram shows the distribution of reciprocation sizes. The scale of the histogram is on the right y axis. Compared to the Interpolating Baseline, the RL agents exclude players that made a sizable reciprocation on the previous trial. In all plots the top row shows data from virtual players and the bottom row shows human data. B and C show the same data for Exp.2 and Exp.3 respectively.

Fig. S6.

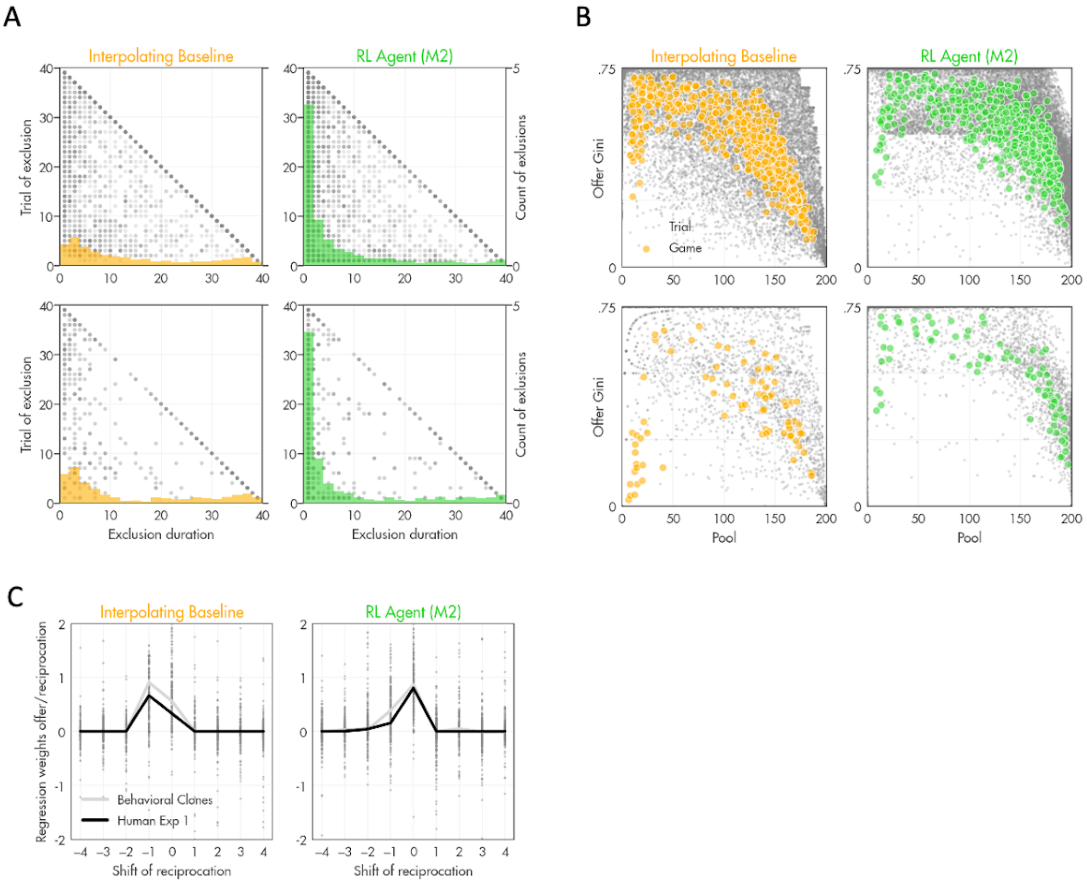

Figure S6. Additional information on Exp. 3. A. Analysis of mechanisms in Exp.3, equivalent to **Fig. 2D.** The duration of each exclusion is plotted against the trial on which reinclusion occurs (dots on the diagonal are "permanent" exclusions that endure until trial 40). The histogram shows the count of exclusions. The top panels are for virtual players and the bottom panels for human players. **B.** The average Gini coefficient of the offer made to players as a function of the pool size, for individual trials (grey dots) and games (coloured dots), both for behavioural clones (upper panels) and human data in Exp.3 (lower panels). See also Fig. 2C. **C.** The offer made by each mechanism to each player as a function of the lagged contribution of that player over adjacent trials. Dots are individual coefficients; black line is the median. See also Fig. 2E.

Fig. S7

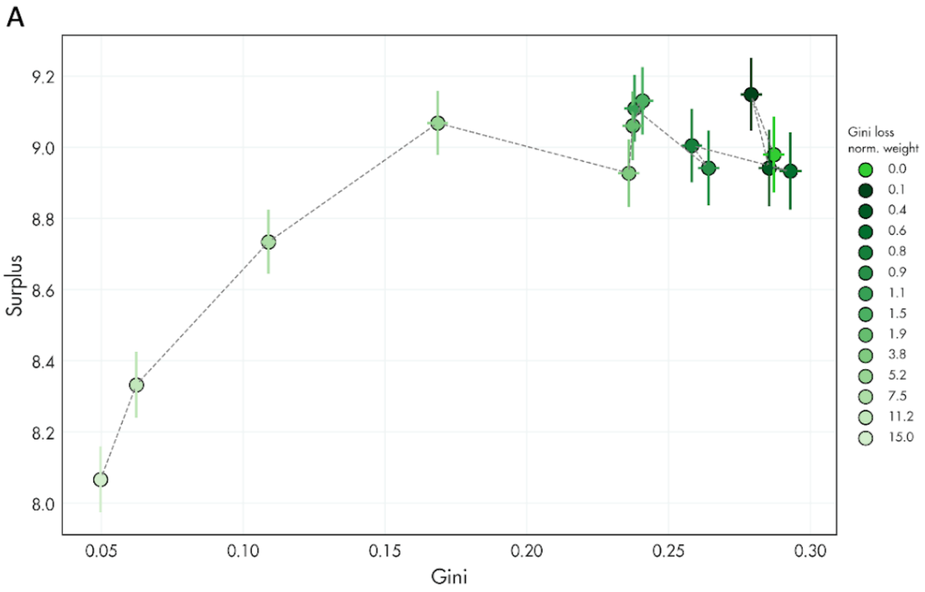

Figure S7. Exploration of additional RL objective. We used virtual players to evaluate the impact of adding an additional objective to the usual reward maximisation of the RL agent. This new objective, called the Gini loss, aims to minimise the Gini coefficient for player surplus and has a scalar weight associated with it. The RL agent (M2) introduced in Exp. 3 corresponds to a Gini loss weight of 0 (saturated green dot). However, as we progressively increase the weight of the Gini loss, we observe a trade-off between minimising the Gini coefficient and maximising the surplus. The legend shows the weight of the Gini loss is normalised to be on the same numerical scale as the reward objective. Each point is the average of 512 games, and standard error bars show 1 S.E.M.

Fig. S8

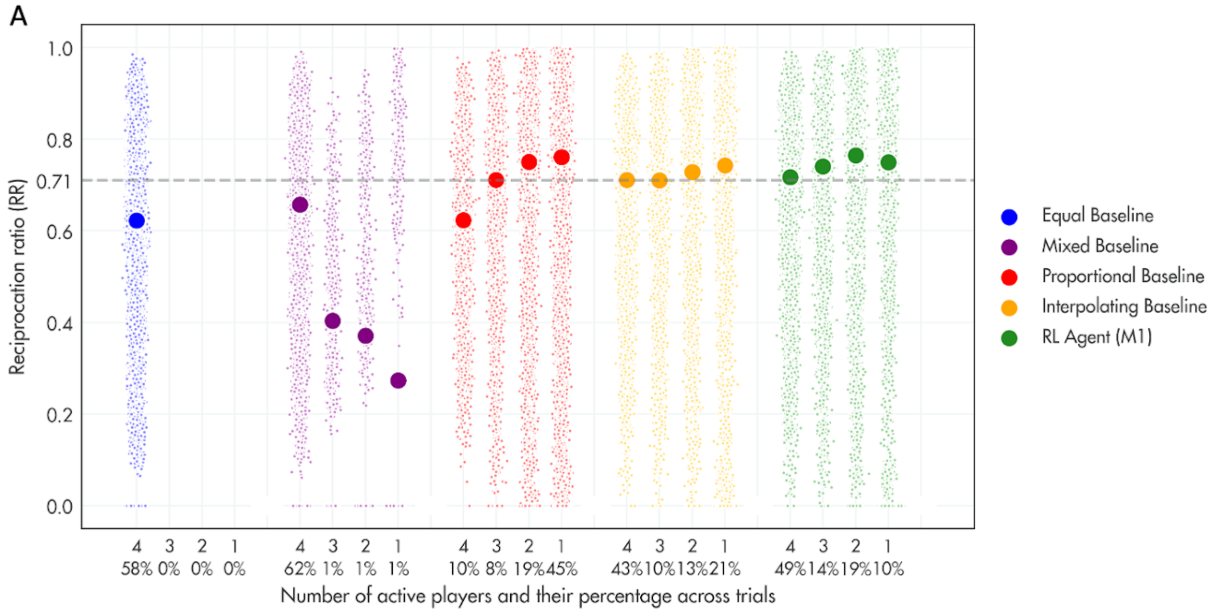

Figure S8. Analysis of mechanism behaviors. In our multiplayer game, the optimal policy for a self-interested player i depends intractably on their assumptions about the future behaviours of other players j. However, for a group to cooperate sustainably (under a constant termination probability) the reciprocation ratio (RR; reciprocation / offer) over all players remaining in the game should average $1/(1+r)$ where r is the growth rate. Here, we plotted the distribution of average RR values for each mechanism as a function of the number of active players. Equal and mixed mechanisms resulted in dramatic under-provisioning of the common pool, as expected. For the other mechanisms, the pool was on average under-provisioned when many active players needed to cooperate, but over-provisioned when a single player sustained the economy. The RL agents (M1 and M2) and interpolating baseline both resulted in approximately optimal RR for the majority of play in which all 4 players remained active. The plot shows the reciprocation ratio for each mechanism, calculated from virtual players, for game periods with differing numbers of active players. Dots are individual trials and crosses are the average of games. The dashed line is the optimal value for r = 0.4.

Fig. S9

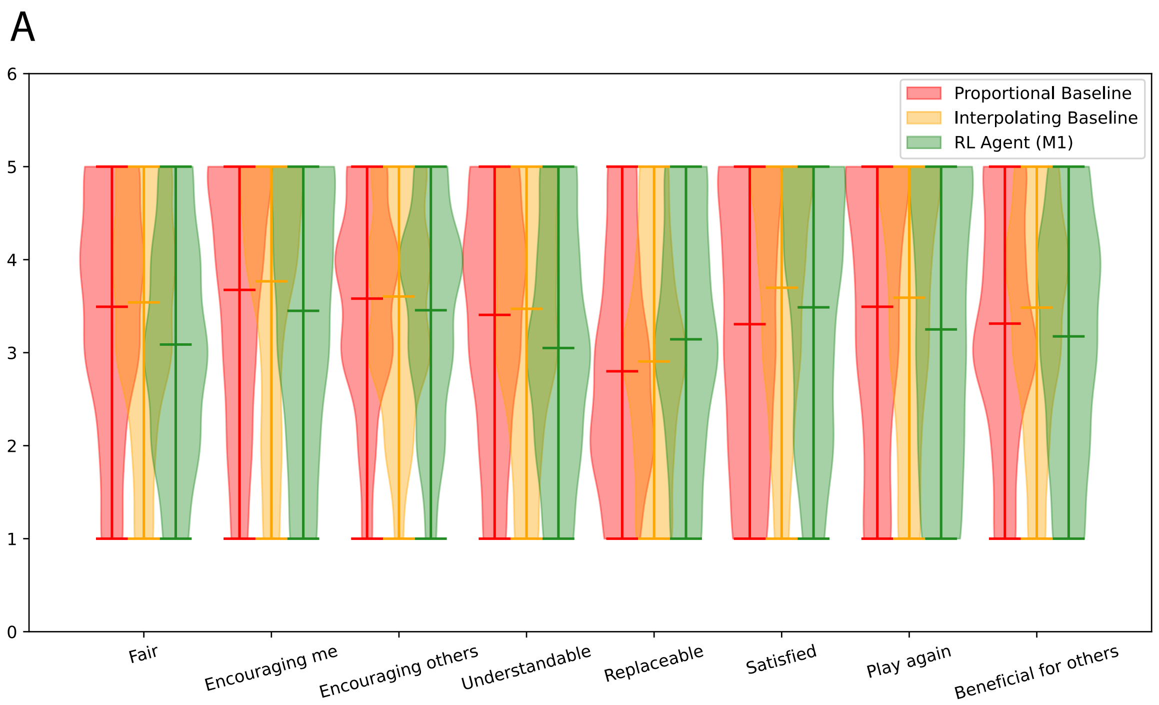

Figure S9. Questionnaire about mechanism preferences in Exp 2. A. Subjective reported preferences for each mechanism in Exp.2. The y-axis shows the average of reports on a Likert scale for a series of questions (see methods).

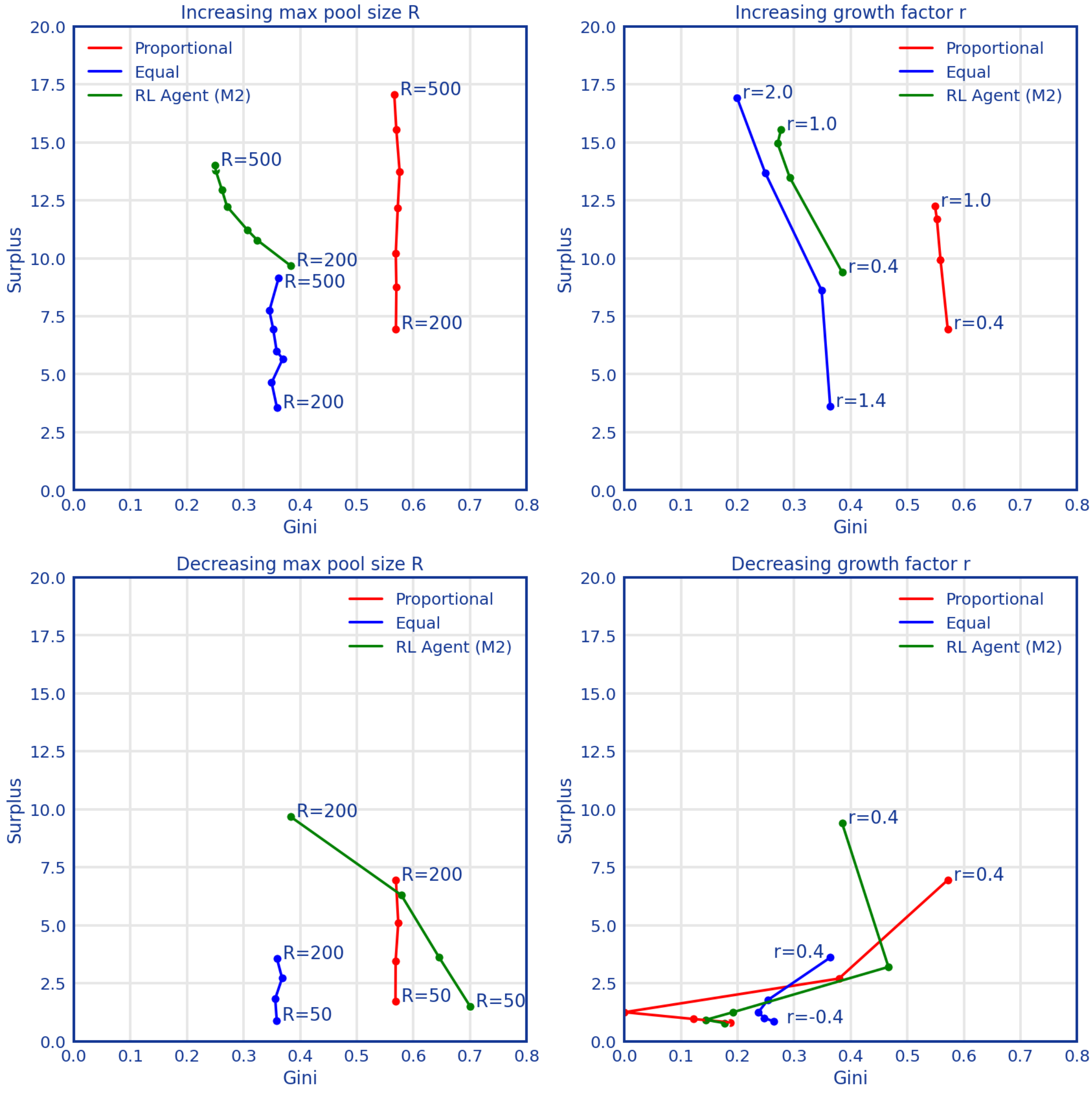

Figure S10. Simulations of different game parameters. Simulation of altering the game parameters R (maximum pool size) and r (growth factor). In the top plots, increasing R or r leads to a decrease in Gini and an increase in surplus, showing that our mechanism extrapolates beyond the trained values (which is surprising, as we only trained with a single value for R and r). When R and r and decreased, effectively constraining the opportunities for collective action, all mechanisms suffer approximately equally. Note that this is a simulation, therefore it also relies on the ability of the virtual players to generalise to the altered game parameters**.**

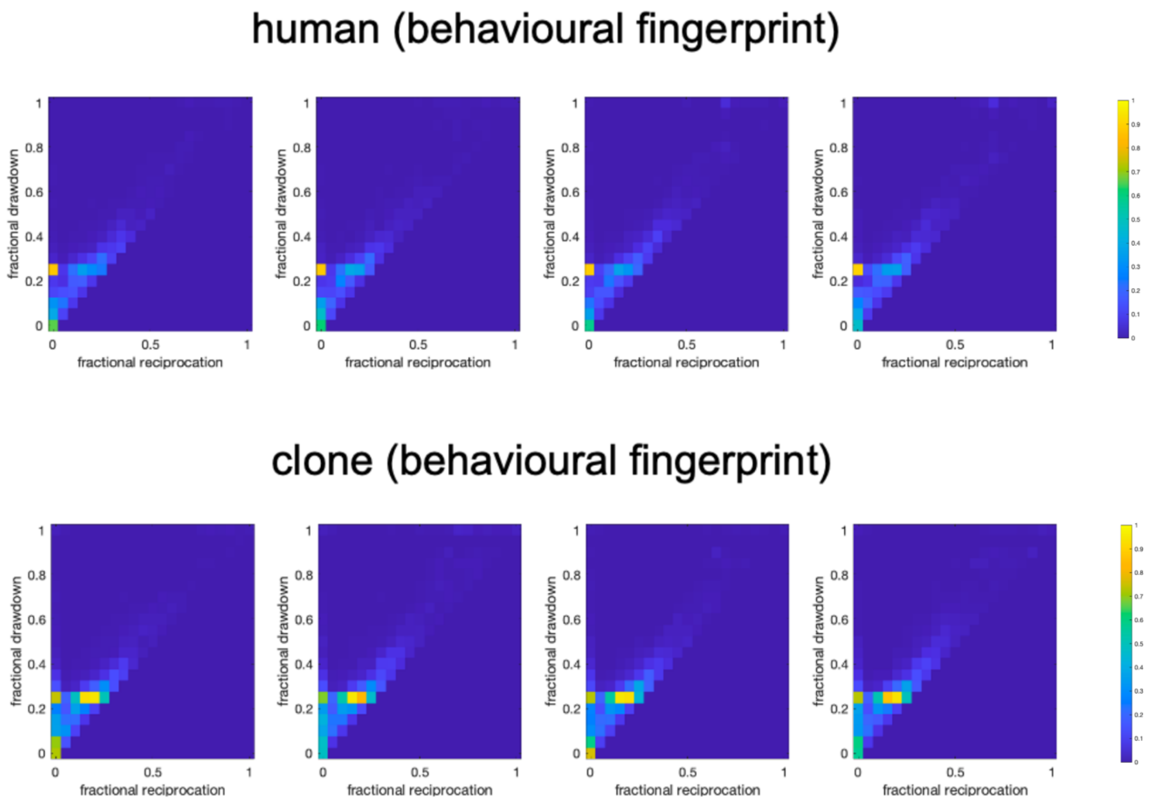

Figure S11. Fit of behavioural cloning. Two-dimensional frequency plot showing fractional reciprocation as a function of the fractional drawdown (endowment) received. The data are shown separately for the humans (here from the training data; but data from all other experiments are similar) and the behavioural clones. The colourmap shows the frequency normalised to unit range. The most salient features of the data are (1) that the data lie above the identity line, because players cannot give back more than they receive; (2) that there is a mode at about 0.25, which is where an equal endowment is offered to all players; and (3) that on average players give roughly in proportion to what they receive. Note that the behavioural clones do a good job of capturing the overall distribution of human behaviour.

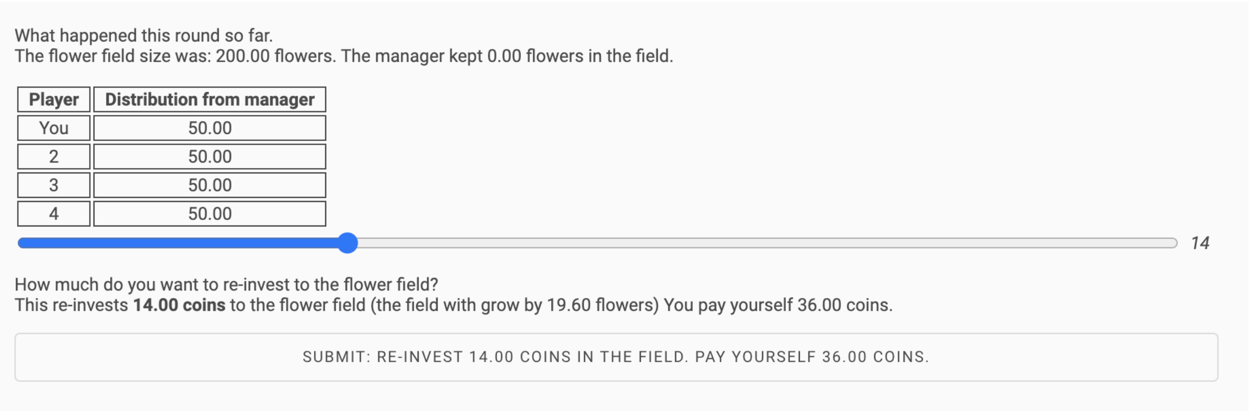

Figure S12. Reciprocation interface.

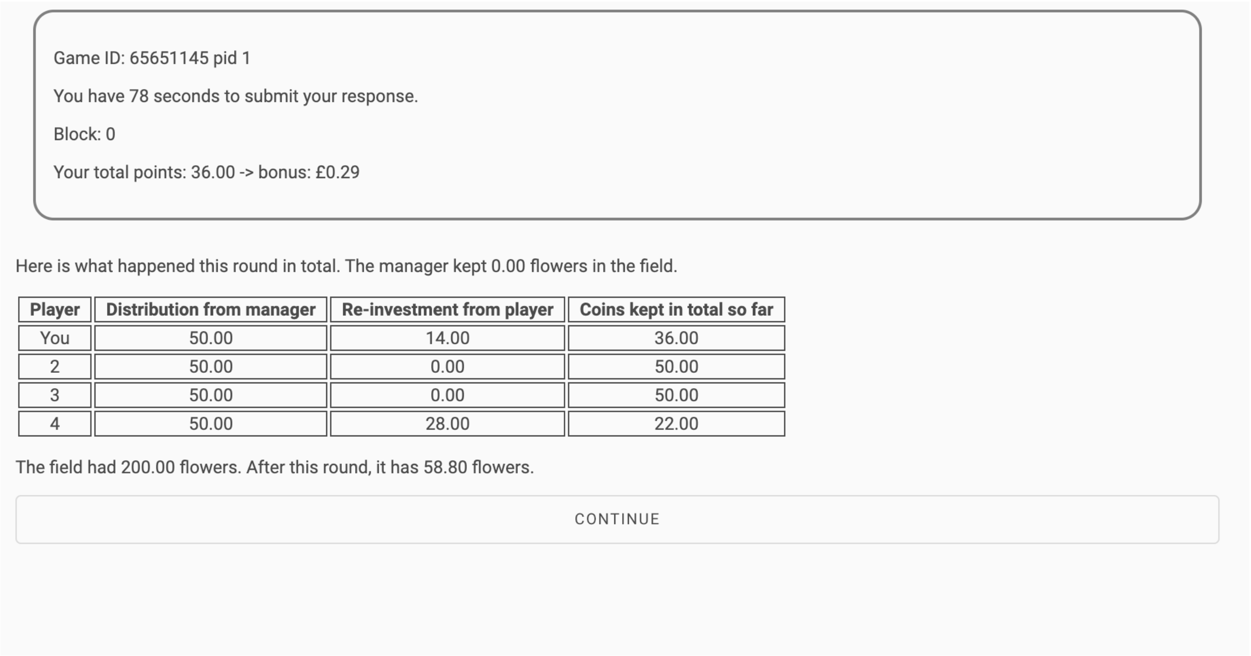

Figure S13. Round overview interface.

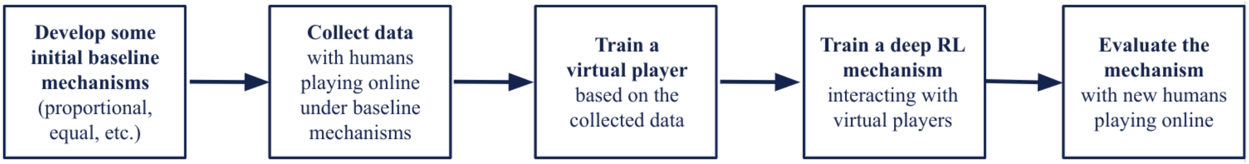

Figure S14. Overview of the Training Pipeline for Mechanism M1.

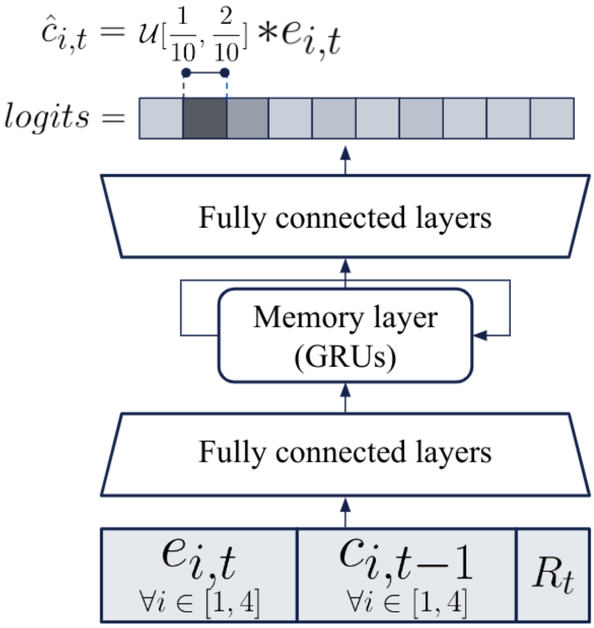

Figure S15. Behavioural clone architecture. Illustration of the neural network architecture used for modelling virtual players (i.e., doing behaviour cloning) to predict a player's contribution at the current step. The network takes as input the observations of each of the

4 players, which includes each of their current endowments offered by the mechanism ($e_{i,t}$), each of their contributions in the previous step ($c_{i,t-1}$), and the current state of the pool (R_t). These features are concatenated and passed through different neural (fully connected and memory-based) layers. Eventually, they are projected into a final 10-dimensional vector (assuming 10 bins), which represents the logits binning the space of possible reciprocations from 0 to the player's current endowment offer. To determine the fraction of reciprocation, a random sampling is performed within the interval defined by the margins of the bin with the highest probability. Multiplying the currently received endowment offer by this fraction yields the predicted absolute value of the reciprocation to be made by the virtual player in the current round ($\hat{c}_{i,t}$).

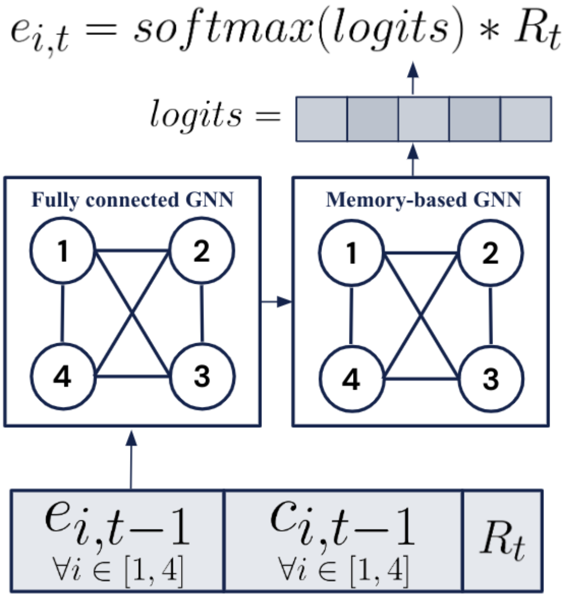

Figure S16. Agent architecture. Illustration of the neural network architecture used for modelling the mechanism's actions at the current step to predict a mechanism's offer to each player at the current step. The network takes as input the observations of each of the 4 players, which includes each of their offered endowments ($e_{i,t-1}$) and contributions ($c_{i,t-1}$) at the previous step, as well as the current state of the pool (R_t). These features are concatenated and passed through different two graph neural networks: one fully connected, the other memory-based. Subsequently, a final linear projection generates a 5-dimensional vector of logits. The softmax over these logits corresponds to the fractions to be offered by the mechanism to each player and be kept in the pool. Multiplying each of these 5 fractions by the current pool resources yields the predicted absolute value of the endowment to be offered by the mechanism to each player at the current step ($e_{i,t}$).

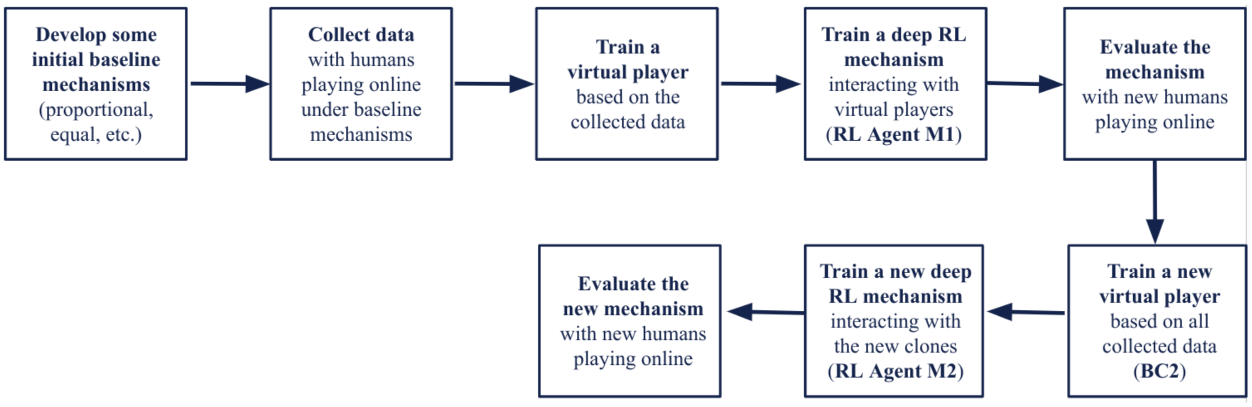

Figure S17. Overview of the Training Pipeline for Mechanism M1.

**Hyper-parameters**	**BC1**	**BC2**
Batch size	256	1024
Total number of updates	700,000	1,500,000
Checkpoint steps	2 BCs at 200,000 and another 2 BCs at step 500,000	1 BC at step 100,000, 1 BC at step 150,000, 5 BCs at step 200,000, and a last BC at step 1,000,000
Learning rate	5e-4 annealed to 5e-6 – exponential decay, with decay rate=0.05 and num_steps_decay_rate=1000	1e-4 annealed to 1e-6 – exponential decay, with decay rate=0.01 and num_steps_decay_rate=1000
Encoder layer	2 non-linear layers with output sizes (16, 32)	2 non-linear layers with output sizes (128, 256, 512)
Memory core	GRU with 64 hidden size	GRU with 512 hidden size
Projection layer	2 non-linear layers with output sizes (32, 16)	2 non-linear layers with output sizes (512, 256, 128)
Output layer	1 linear layer with output size (10,)	1 linear layer with output size (10,)
Activations	tanh	tanh

Table S1: Hyperparameters used for virtual players (or BC) training.

**Hyper-parameters**	**M1**	**M2**

Batch size	256	1024
Total number of updates	500,000	800,000
Checkpoint step	300,000	450,000
Learning rate	1e-3 annealed to 1e-5 (exponential decay, with decay rate=0.05 and num_steps_decay_rate=1000)	1e-5 (fixed)
First GNN	Edges: 1 fully connected layer with output size (32,).<br>Nodes: 1 fully connected layer with output size (32,).<br>Globals: 1 fully connected layer with output size (32,).	Edges: 1 fully connected layer with output size (32,).<br>Nodes: 1 fully connected layer with output size (32,).<br>Globals: 1 fully connected layer with output size (32,).
Second GNN	Edges: 1 fully connected layer with output size (32,).<br>Nodes: GRU with 32 hidden size, followed by 1 non-linear layer with output size (32,), and 1 linear layer with output size (1,).<br>Globals: 2 fully connected layers with output size (32, 1).	Edges: 1 fully connected layer with output size (32,).<br>Nodes: GRU with 64 hidden size, followed by 1 non-linear layer with output size (32,), and 1 linear layer with output size (1,).<br>Globals: 2 fully connected layers with output size (32, 1).
Activations	ReLU	ReLU

Table S2: Hyperparameters used for mechanism training.
